# New case of HIV cure: joined forces of haploidentical stem cells and HLA-mismatched cord blood

**DOI:** 10.1038/s41392-023-01514-4

**Published:** 2023-06-16

**Authors:** David Peterhoff

**Affiliations:** Institute for Clinical Microbiology and Hygiene, University Hospital Regensburg, Regensburg, Germany

**Keywords:** Infectious diseases, Outcomes research

Recently, Hsu et al*.* reported the remission and possible cure of HIV-1 infection in the context of an allogeneic hematopoietic stem cell transplant for the treatment of acute myeloid leukemia (AML).^1^ This is the fourth case of a potential cure for HIV infection ever, the first in a woman, the first in a person of mixed race, and the first in which a CCR5Δ32/Δ32 haplo-cord transplant was used.

According to UNAIDS epidemiological estimates for 2022, 38.4 million people are currently living with HIV. After infection, the virus persists in latent reservoirs of resting CD4^+^ T memory cells for a lifetime. With antiretroviral therapy (ART), the viral load can be reduced below the detection limit, which allows people living with HIV (PLWH) to lead a life largely unaffected by the disease.

**Fig. 1:**
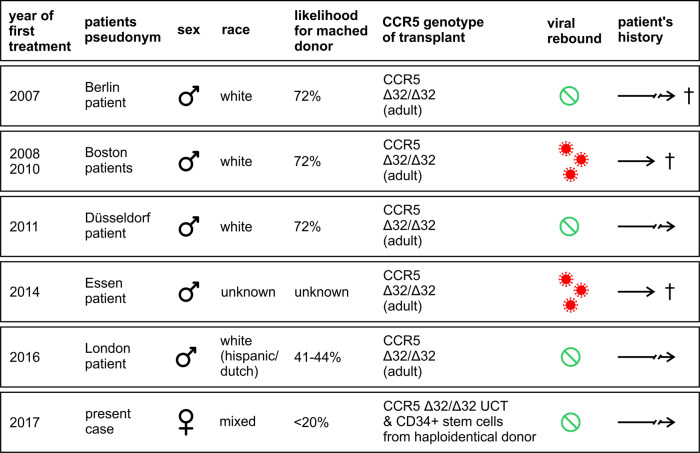
HIV-1 individual curative trials. In all listed cases, with the exception of the two “Boston patients” and the “Essen patient”, a cure of the HIV-1 infection can be assumed so far. The “Berlin patient” succumbed to a relapse of his AML 12 years after his treatment. In general, the effectiveness of HIV-1 cure by stem cell transplantation is difficult to assess because, on the one hand, patients often succumb to complications related to SCT (and consequently the cure of the infection cannot be shown) and, on the other hand, it is possible that some failed attempts have not been published. The likelihood of finding a HLA matched adult unrelated donor is given without regard to the CCR5 genotype. WT wild type, Δ32 CCR5 frameshift mutation, UCT umbilical cord blood transplant

To date, three cases of HIV cure have been described (Fig. 1). In all cases, acute myeloid leukemia (AML) was treated by using an unrelated homozygous CCR5Δ32/Δ32 allograft. Homozygous carriers of the Δ32 deletion in HIV’s co-receptor CCR5 (0.8–1% individuals of northern European descent) are resistant to the predominantly transmitted CCR5 using (“R5-tropic”) strains. Thus, no rebound of infection from remaining reservoirs was detectable even when ART was interrupted due to the newly established resistance.

The number of potential adult donors for haematopoietic cell transplantation is relatively small because a very close match of the human leukocyte antigen (HLA) is required for 8 out of 8 or 7 out of 8 alleles. In contrast, transplantation of stem cells from the umbilical cord (UCT) does not require such a strict HLA match between donor and recipient.^2^ Furthermore, it causes little graft-versus-host disease (GvHD) and less disease recurrence. However, limited numbers of progenitor cells in umbilical cord blood may result in delayed and unpredictable recovery. Moreover, adoptive donor T cell transfer for treatment of viral infections or relapse after transplantation is not available.

Hsu et al. now used an improved variant of the UCT, where in parallel CD34^+^ cells from a haploidentical donor were transplanted.^1^ Supportive co-transplantation of purified peripheral blood CD34^+^ cells (CD34 is a marker for hematopoietic stem cells) from an HLA-haploidentical donor leads to an early recovery of neutrophils and thus closes the gap between pretransplant conditioning (eradicating residual leukemic cells and suppressing host immunity to prevent rejection) and engrafting of the UCT cells which reduces the risk for infectious complications caused by protracted neutropenia.

To test the potential of haplo-cord stem cell transplantation for HIV cure, the present observational study enrolled HIV-1-infected individuals who required allogeneic stem cell transplantation due to an underlying malignant disease. Two HIV-1 positive middle-aged patients were treated: a man who developed Hodgkin’s lymphoma and a woman who developed AML. Both patients underwent a haplo-cord transplant with CCR5Δ32/Δ32 cord blood units. The man lost the CCR5Δ32/Δ32 graft and died of recurrent Hodgkin’s lymphoma within a year. In case of the woman, the transplantation process was uncomplicated. Initially, two weeks after treatment, 82% of her lymphatic and 98% of her myeloid lineages were derived from the haploidentical donor, while after 14 weeks 100% engraftment with the umbilical cord cell lineage was reported. Of note, comparable to the “Berlin patient”, the patient in the present case received whole-body irradiation in addition to chemotherapy for conditioning, which was not used in the “London” and “Düsseldorf” patients to adjust for the increased rejection risk due to HLA-disparity.^3^ After a period of 3 years with undetectable HIV RNA levels, the patient decided to stop ART. HIV-1 RNA levels remained undetectable in the further course, indicating HIV-1 remission and a possible cure.

The authors draw particular attention to the implications of their study for racial health equity, as the likelihood of finding an HLA-matched donor is lower in individuals of diverse ancestry. The less stringent match requirements in UCT extends the circle of possible recipients of CCR5Δ32/Δ32 stem cell therapy in PLWH. The authors furthermore emphasize that their patient never developed GvHD. GvHD was considered critical for HIV-1 cure in the Berlin patient due to the underlying graft-versus-leukemia reaction which also eradicates residual host T cells potentially infected with HIV (“graft versus HIV effect”). In the present case, however, GvHD was not a prerequisite for the potential cure, and the optimized UCT method therefore appears to be particularly safe, without the undesirable sequelae of GvHD. This is in line with results from a phase II trial combining haploidentical peripheral stem cells and HLA-mismatched UCT showing a low relapse rate despite lack of clinical symptoms of graft-versus-host disease in a significant proportion of patients.^4^

In the case of the “Essen patient”, the cure of the HIV-1 infection failed, as a rebound of previously undetected CXCR4-tropic virus occurred after transplantation with adult CCR5Δ32/Δ32 stem cells. In the current case, the autologous virus was exclusively CCR5-tropic and thus could not infect the engrafted CCR5Δ32/Δ32 cells. Unexpectedly the cells were also not susceptible to a CXCR4-tropic laboratory strain in vitro. This has not been observed in adult CCR5Δ32/Δ32 stem cell transplantation and constitutes another benefit of UCT which could be of advantage in cases like the “Essen patient”. For the current case, it is to be hoped that X4-tropic viruses do not rebound from non-eliminated reservoirs in the course of an extended observation period.

So far so very good—yet it seems that the present case is simply another case of blessing in disguise. So what are the current prospects for curative HIV therapy? Likely, modern immunotherapy approaches represent a less risky path to HIV cure for PLWH but without severe secondary illnesses.^5^ Therapeutic concepts based on adoptive cell transfer (ACT) include the application of chimeric antigen receptor T cells, which can recognize and eliminate HIV-infected cells. However, such therapies likely require the use of latency reversing agents, which can have toxic side effects. ACT also opens up the possibility of direct manipulation of the isolated T cells using genome editing (e.g., via CRISPR–Cas9), either to directly target the integrated provirus or to generate resistant cells (e.g., by introducing the CCR5Δ32 deletion), but are challenging because of the risk of off-target effects. Thus, despite encouraging progress with newer curative strategies, questions of safety and efficacy remain challenging.

In summary, with their UCT-based therapy, Hsu et al. have successfully demonstrated the feasibility of an alternative strategy for HIV-1 cure, facilitating the inclusion of mixed-race patients, informing further developments in the field of gene and cell therapy, and raising hopes for more cases of long-term-remission and cure.
